# Supplementary material for: Complex‐centric proteome profiling by SEC‐SWATH‐MS
Source: Mol Syst Biol. 2019 Jan 14;15(1):e8438. doi: 10.15252/msb.20188438 (PMC6346213; doi:10.15252/msb.20188438)
Supplement: Supplementary file 7 — Dataset EV6 [file MSB-15-e8438-s007.zip › feature_plots_bioplex/Q9Y580.pdf]

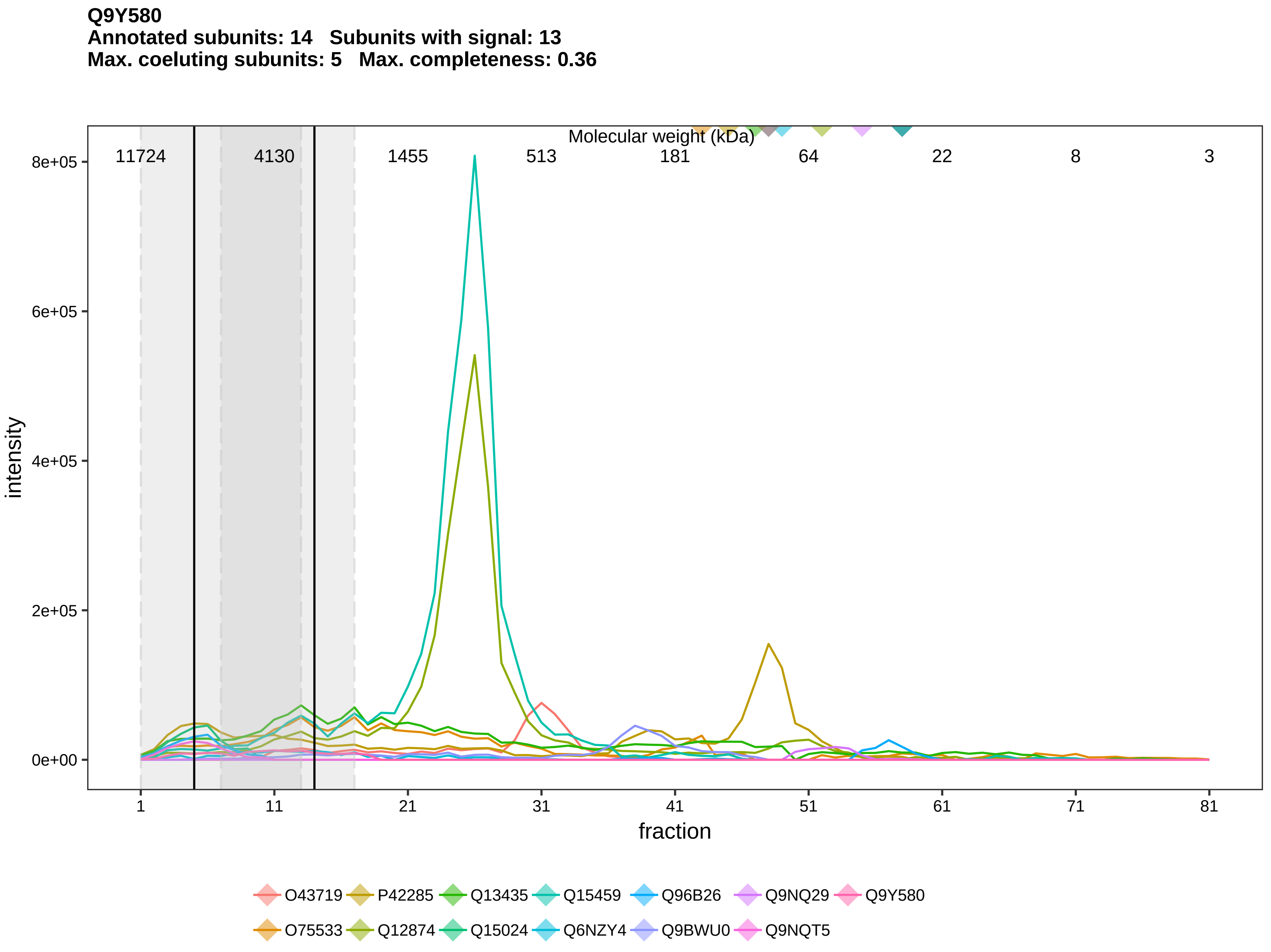

Q9Y580
Annotated subunits: 14 Subunits with signal: 13
Max. coeluting subunits: 5 Max. completeness: 0.36
Molecular weight (kDa)
11724
4130
1455
513
181
64
22
8
3
intensity
8e+05
6e+05
4e+05
2e+05
0e+00
fraction
1
11
21
31
41
51
61
71
81
O43719
P42285
Q13435
Q15459
Q96B26
Q9NQ29
Q9Y580
O75533
Q12874
Q15024
Q6NZY4
Q9BWU0
Q9NQT5
